# Concizumab prophylaxis in people with hemophilia A or B without inhibitors: patient-reported outcome results from the phase 3 explorer8 study

**DOI:** 10.1016/j.rpth.2025.102705

**Published:** 2025-02-20

**Authors:** Pantep Angchaisuksiri, Sylvia von Mackensen, Shashikant Apte, Gary Benson, Hermann Eichler, Amy Findley, Tadashi Matsushita, Camila M. Mazini Tavares, Morten Puggaard Ravn, Jameela Sathar, Laura Villarreal Martinez, Guy Young

**Affiliations:** 1 Division of Haematology, Department of Medicine, Ramathibodi Hospital, Mahidol University, Bangkok, Thailand; 2 Department of Medical Psychology, University Medical Centre Hamburg-Eppendorf, Hamburg, Germany; 3 Department of Haematology, Sahyadri Specialty Hospitals, Pune, India; 4 Department of Haematology, Belfast Health and Social Care Trust, Belfast, Northern Ireland, United Kingdom; 5 Institute of Clinical Haemostaseology and Transfusion Medicine, Saarland University and University Hospital, Homburg, Germany; 6 Medical & Science Patient Focused Drug Development, Novo Nordisk A/S, Søborg, Denmark; 7 Department of Transfusion Medicine, Nagoya University Hospital, Nagoya, Japan; 8 Medical & Science Rare Bleeding Disorders, Novo Nordisk A/S, Søborg, Denmark; 9 Biostatistics RD & AT 1, Novo Nordisk A/S, Søborg, Denmark; 10 Department of Haematology, Ampang Hospital, Kuala Lumpur, Malaysia; 11 Department of Haematology, Dr. José Eleuterio González Monterrey University Hospital, Monterrey, Nuevo León, México; 12 Hemostasis and Thrombosis Centre, Children’s Hospital of Los Angeles, Keck School of Medicine, University of Southern California, Los Angeles, California, USA

**Keywords:** concizumab, health-related quality of life, hemophilia A, hemophilia B, patient-reported outcomes

## Abstract

**Background:**

Patient-reported outcomes (PROs) can provide useful insights into patient perception of concizumab, an anti–tissue factor pathway inhibitor monoclonal antibody intended for once-daily, subcutaneous prophylaxis for hemophilia A (HA) or hemophilia B (HB), with and without inhibitors.

**Objectives:**

To evaluate PROs from the phase 3 explorer8 study (NCT04082429).

**Methods:**

Male patients aged ≥12 years with HA/HB without inhibitors were enrolled and randomized 1:2 to no prophylaxis/on-demand treatment (arm 1) or concizumab prophylaxis (arm 2) or allocated to concizumab prophylaxis (arms 3 and 4). PRO questionnaires included the 36-item short-form health survey version 2, Haemophilia Quality of Life Questionnaire for Adults, Hemophilia Treatment Experience Measure, and Haemophilia Patient Preference Questionnaire.

**Results:**

Estimated treatment difference for change in 36-item short-form health survey version 2 “bodily pain” and “physical functioning” from baseline to week 24 between patients in arms 1 and 2 was 9.5 points (95% CI, 2.4 to 16.7) and 0.3 points (95% CI, −5.1 to 5.6), respectively. Estimated treatment difference at week 24 between patients in arms 1 and 2 was −18.0 points (95% CI, −26.4 to −9.5) for Haemophilia Quality of Life Questionnaire for Adults “total score” and −16.8 points (95% CI, −32.2 to −1.4) for “physical health.” Hemophilia Treatment Experience Measure and Haemophilia Patient Preference Questionnaire results favored concizumab prophylaxis over no prophylaxis or previous treatment.

**Conclusion:**

PRO data from the phase 3 explorer8 study provided additional support for concizumab prophylaxis compared with no prophylaxis as a treatment option for patients with HA/HB.

## Essentials

• Concizumab is a hemophilia treatment injected once daily under the skin.
• People with hemophilia and no inhibitors answered health questionnaires while on concizumab.
• Health-related quality of life improved and treatment burden reduced with concizumab treatment.
• Seventy-one percent of patients preferred concizumab over their previous hemophilia treatment.

## 1 Introduction

Patient-reported outcomes (PROs) provide added value to clinical study interpretation by providing insights into patient experience and perception of treatment [1,2]. PRO is an umbrella term encompassing various types of patient-reported endpoints that fall under multiple constructs such as perceived symptoms, functional status, health perception, and quality of life [3,4]. Commonly described PROs in hemophilia include instruments assessing health-related quality of life (HRQoL), treatment experience, and treatment burden [[5], [6], [7]]. For people with hemophilia, HRQoL is often compromised due to pain or impact on physical functioning [8]. In addition, the burden of treatment may also affect HRQoL of people with hemophilia [8]. Evaluating PROs in these patients provides a deeper understanding of the impact of new treatments beyond clinical benefits from a patient perspective [1,2].

In general, the standard of care for people with hemophilia involves prophylactic treatment to replace the deficient coagulation factor [9]. Factor replacement therapies are often administered by intravenous infusions, which can be associated with considerable treatment burden [[10], [11], [12]]. Inhibitor formation is a treatment-associated challenge that leads to suboptimal efficacy [13,14]. Nonfactor replacement therapies that can be administered subcutaneously are available and may alleviate treatment burden [15,16]. However, significant unmet needs persist, especially for people with hemophilia B (HB). To date, there is no licensed prophylactic treatment for patients with HB with inhibitors, while additional treatment options that enable individualization of prophylactic care for patients with HB without inhibitors would further improve outcomes in these patients.

Concizumab is a humanized anti–tissue factor pathway inhibitor monoclonal antibody intended for prophylaxis in people with hemophilia A (HA) or HB with and without inhibitors, and is currently approved by the European Medicines Agency and the US Food and Drug Administration for once-daily, subcutaneous prophylaxis in HA or HB with inhibitors [17,18]. In Japan, concizumab is approved for prophylaxis in HA or HB with and without inhibitors [19]. It is a new class of monoclonal antibodies that acts by preventing inhibition of coagulation by tissue factor pathway inhibitor, thereby restoring thrombin generation and leading to a reduction in bleeding episodes in people with hemophilia [[20], [21], [22]]. Daily subcutaneous prophylactic treatment with concizumab has been shown to reduce bleeding episodes in people with HA or HB, with or without inhibitors, compared with no prophylaxis (on-demand treatment) [23,24]. Concizumab could offer an additional therapeutic option for individualization of prophylactic care in patients with HB without inhibitors, as well as a prophylactic option for HB with inhibitors, a population for whom there is still no efficient treatment currently available.

Here, we report PRO results from explorer8, a phase 3 study investigating the efficacy and safety of once-daily subcutaneous concizumab prophylaxis in people with HA or HB without inhibitors.

## 2 Methods

### 2.1 Study design and patient population

**Figure 1:**
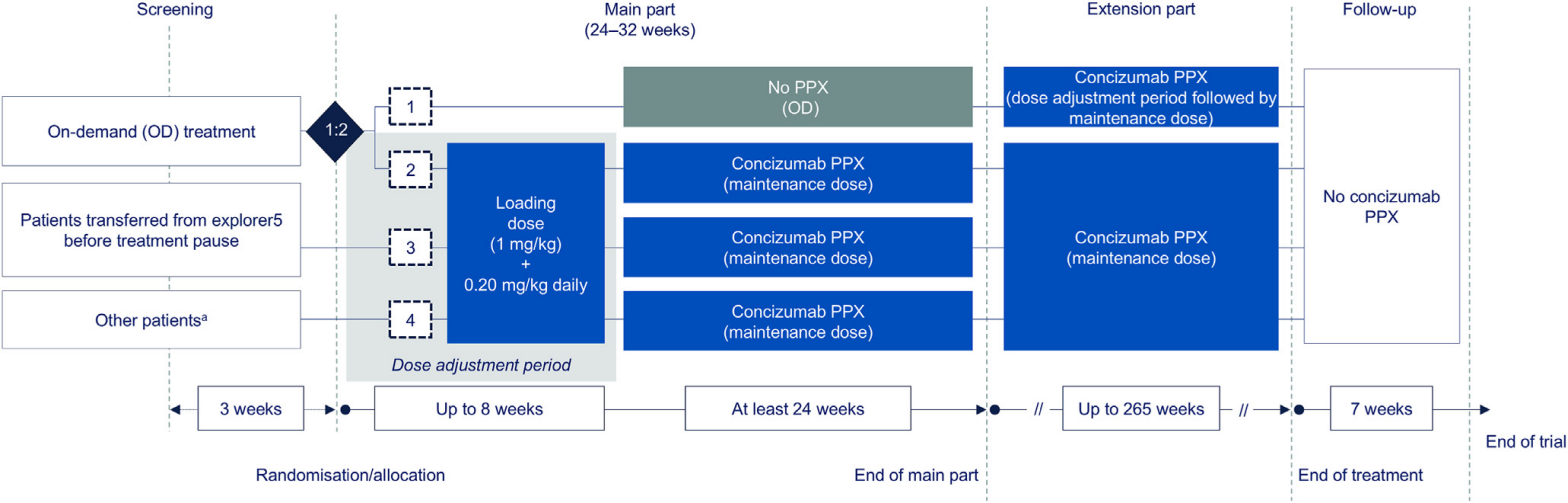
explorer8 study design. [24] ^a^Additional patients on on-demand (OD) treatment or patients on prophylaxis (PPX) with factor replacement, patients from explorer5 (phase 2 concizumab study on people with hemophilia A; NCT03196297) [18] enrolled after the restart and patients randomized to arms 1 or 2 before the treatment pause.

explorer8 is a prospective, multicenter, open-label, phase 3a clinical study conducted across 31 countries (ClinicalTrials.gov identifier: NCT04082429) [24]. Male persons aged ≥12 years with severe HA (factor VIII levels <1%) or moderate/severe HB (factor IX levels ≤2%) weighing >25 kg and with documented coagulation factor-containing treatment in the 24 weeks prior to screening (if not transferring from explorer5; NCT03196297 [25]; Figure 1) were included in the study. Exclusion criteria, detailed in the Supplementary Methods, include known or suspected hypersensitivity to any constituent of concizumab; previous participation in any clinical trial of investigational medicinal products within 5 half-lives or 30 days from screening, whichever is longer; and history, clinical signs, or treatment of thromboembolic disease.

Patients were randomized 1:2 to no prophylaxis (arm 1) or concizumab prophylaxis (arm 2) or assigned to nonrandomized concizumab prophylaxis (arms 3 and 4) (Figure 1). A concizumab treatment pause was implemented in March 2020 following the occurrence of nonfatal thromboembolic events in 3 patients receiving concizumab in explorer8 (*n* = 2) and explorer7 (*n* = 1; NCT04083781) [24]. Following thorough investigations, concizumab treatment resumed with risk mitigation measures, including a new dosing regimen and breakthrough bleed management guidance (please see the Supplementary Methods for further details of this pause). With the new concizumab dosing regimen, patients received an initial loading dose of 1.0 mg/kg on day 1 followed by a daily dose of 0.20 mg/kg from day 2 during the dose adjustment period.

The daily concizumab maintenance dose could be decreased to 0.15 mg/kg (if concizumab plasma concentration was >4000 ng/mL), increased to 0.25 mg/kg (if concizumab plasma concentration was <200 ng/mL) or maintained at 0.20 mg/kg based on concizumab plasma concentration measurement following 4 weeks of treatment. After the treatment pause, new patients were randomized to arm 1 (no prophylaxis) and arm 2 (concizumab prophylaxis), with patients randomized prior to the treatment pause moving to arm 4. Patients who were allocated to arms 3 and 4 before the pause re-entered the arm they were initially allocated to.

The study was approved by independent ethics committees/institutional review boards and conducted in accordance with the Declaration of Helsinki and the International Council for Harmonisation of Technical Requirements for Pharmaceuticals for Human Use Guideline for Good Clinical Practice. An independent data monitoring committee reviewed and evaluated the data at predefined time points and on an ad hoc basis to provide study conduct recommendations for the safeguarding of patients. Written informed consent was obtained before the conduct of any study-related activity.

### 2.2 Objectives and endpoints

The primary objective of the explorer8 study was to compare the effect of concizumab prophylaxis with no prophylaxis in reducing the number of bleeding episodes in adult and adolescent patients with HA/HB [24]. The primary endpoint was the number of treated spontaneous and traumatic bleeding episodes during the main part of the study (24-32 weeks).

The secondary objective was to compare the efficacy of concizumab vs previous prophylaxis in reducing number of bleeding episodes, assessed by the number of treated spontaneous and traumatic bleeding episodes.

Exploratory objectives were to compare PROs between patients who received concizumab prophylaxis vs no prophylaxis and to explore patients’ treatment preferences for no prophylaxis, concizumab prophylaxis, or previous prophylaxis. Exploratory PRO endpoints included change in HRQoL, treatment burden, and treatment-related preferences.

Results for the explorer8 primary and secondary objectives have been previously published and will not be discussed in this article [24]. Here, results for the exploratory objectives on PROs are presented.

### 2.3 PRO questionnaires

PRO questionnaires were used to assess HRQoL, treatment burden, and treatment preference of patients in explorer8.

#### 2.3.1 HRQoL

HRQoL of patients with HA/HB in explorer8 was assessed with the 36-item short-form health survey version 2 (SF-36v2), a generic PRO questionnaire, and the Haemophilia Quality of Life Questionnaire for Adults (Haem-A-QoL), a hemophilia-specific questionnaire. The SF-36v2 and Haem-A-QoL questionnaires were completed at baseline and weeks 4, 8, 16, and 24. Comparisons between patients on concizumab prophylaxis and no prophylaxis were made based on available data from patients aged ≥17 years.

The SF-36v2 questionnaire with a 4-week recall period measures an individual’s overall HRQoL across 8 health scales (bodily pain, physical functioning, role-physical, general health, vitality, social functioning, role-emotional, mental health); 2 component summary scores can be calculated (physical component summary and mental component summary scores) [26,27]. Higher scores indicate better HRQoL. The SF-36v2 scores are norm-based, and scores were transformed to a scale with a mean of 50 and a standard deviation of 10 (based on data from the US general population) [28]. Key exploratory endpoints were change from baseline to week 24 in SF-36v2 “bodily pain” and “physical functioning.”

The Haem-A-QoL captures the physical, emotional, and social components across 10 domains (physical health, feeling, view of yourself, sport and leisure, work and school, dealing with hemophilia, treatment, partnership and sexuality, family planning and future) [29,30]. In the Haem-A-QoL questionnaire, lower domain and total scores correspond to better HRQoL. Key exploratory endpoints were change from baseline to week 24 in Haem-A-QoL “total score” and “physical health.”

#### 2.3.2 Patient-Reported Outcomes Measurement Information System

The Patient-Reported Outcomes Measurement Information System (PROMIS) numeric rating scale v1.0 pain intensity 1a was used to support the evaluation of pain intensity from SF-36v2 (“bodily pain” scale) [31,32]. Patients were asked to rate their average pain in the past 7 days on a scale from 0 (no pain) to 10 (worst imaginable pain). Higher scores indicate greater pain intensity. The PROMIS short-form v2.0 upper extremity 7a was used to support the assessment of SF-36v2 “physical functioning” [31,32]. Patients were asked to respond to 7 questions on physical functioning in the upper extremities with a scale ranging from “without any difficulty” to “unable to do.” Higher scores indicate a higher level of physical functioning.

#### 2.3.3 Patient Global Impression of Severity and Patient Global Impression of Change

Patients completed both Patient Global Impression of Severity (PGI-S) and Patient Global Impression of Change (PGI-C) questionnaires on physical functioning at baseline and at weeks 4, 8, 16, and 24. In the PGI-S questionnaire on physical functioning, patients were asked to choose the response that best describes their level of physical functioning over the past week from 5 options, “very good,” “good,” “fair,” “poor,” and “very poor” [33]. In the PGI-C questionnaire on physical functioning, patients were asked to choose the response that best describes their overall change in level of physical functioning after starting treatment from a response scale with 7 options ranging from “very much better” to “very much worse” [33].

#### 2.3.4 Treatment burden

The Hemophilia Treatment Experience Measure (Hemo-TEM) questionnaire contains questions related to treatment experience within the domains “injection difficulties,” “physical impact,” “interference,” “treatment bother,” and “emotional impact” [34]. Patients completed the Hemo-TEM questionnaire at baseline and at week 24. Lower scores in all Hemo-TEM domains indicate lower treatment burden. The exploratory endpoint was the change from baseline to week 24 in Hemo-TEM “total score.” Treatment differences between arm 1 and arm 2 at week 24 were estimated.

#### 2.3.5 Treatment preference

The ad hoc Haemophilia Patient Preference Questionnaire (H-PPQ) was created to assess treatment preference in the concizumab explorer clinical studies and consists of 3 questions that assess several aspects of patients’ treatment-related preference, including the reasons and the strength of their preferences [23,33]. At week 24, patients were asked to complete the H-PPQ. In addition to the full study population, H-PPQ results were explored in a subset of patients in arm 4 who had been on a stable prophylaxis regimen for at least 24 weeks in explorer6 (NCT03741881) and who entered the maintenance period in explorer8.

### 2.4 Statistical analysis

The mixed model for repeated measures (MMRM) was used to evaluate changes in the SF-36v2 and Haem-A-QoL scores from baseline to week 24 for patients in arms 1 and 2, with treatment, type of hemophilia, and bleeding frequency (<9 or ≥9 bleeding episodes during the past 24 weeks prior to screening) as factors and the baseline score as a covariate, all nested within week (weeks 4, 8, 16, and 24). An unstructured covariance matrix was used to describe the variability for the repeated measurements for a patient. The MMRM allows for data analysis with baseline and at least 1 additional postbaseline value.

An analysis of covariance was applied to evaluate the change in Hemo-TEM scores from baseline to week 24 for patients in arms 1 and 2, with treatment, type of hemophilia, and bleeding frequency prior to screening as factors and the baseline value as a covariate.

Data from the H-PPQ questionnaire are presented as number and percentage of patients with responses in each category.

### 2.5 Meaningful within-patient change

Post hoc and exploratory analyses of meaningful within-patient change (MWPC) for SF-36v2 “physical functioning” and Haem-A-QoL “physical health” at week 24 were performed to determine the proportion of patients in arms 1 and 2 achieving the MWPC thresholds. PGI-S on physical functioning was used as an anchor measure based on 1-category changes.

## 3 Results

### 3.1 Study population

The study population of explorer8 has been described previously, including baseline characteristics. Briefly, a total of 148 patients were randomized or allocated to no prophylaxis (arm 1, *n* = 21) or concizumab prophylaxis (arm 2, *n* = 42; arm 3, *n* = 9; arm 4, *n* = 76) after treatment restart [24].

### 3.2 PRO questionnaires

Results from the PRO questionnaires are presented as combined analyses of data from patients with HA/HB. Analyses by hemophilia subtype (HA and HB) are available in Supplementary Figure S2 (SF-36v2), Supplementary Figure S4 (Haem-A-QoL), Supplementary Figure S6 (Hemo-TEM), and Supplementary Figure S7 (H-PPQ in a subset of arm 4 patients who had been on stable prophylaxis previously). The number of respondents for each PRO questionnaire is presented in Supplementary Table S1.

#### 3.2.1 HRQoL

**Figure 2:**
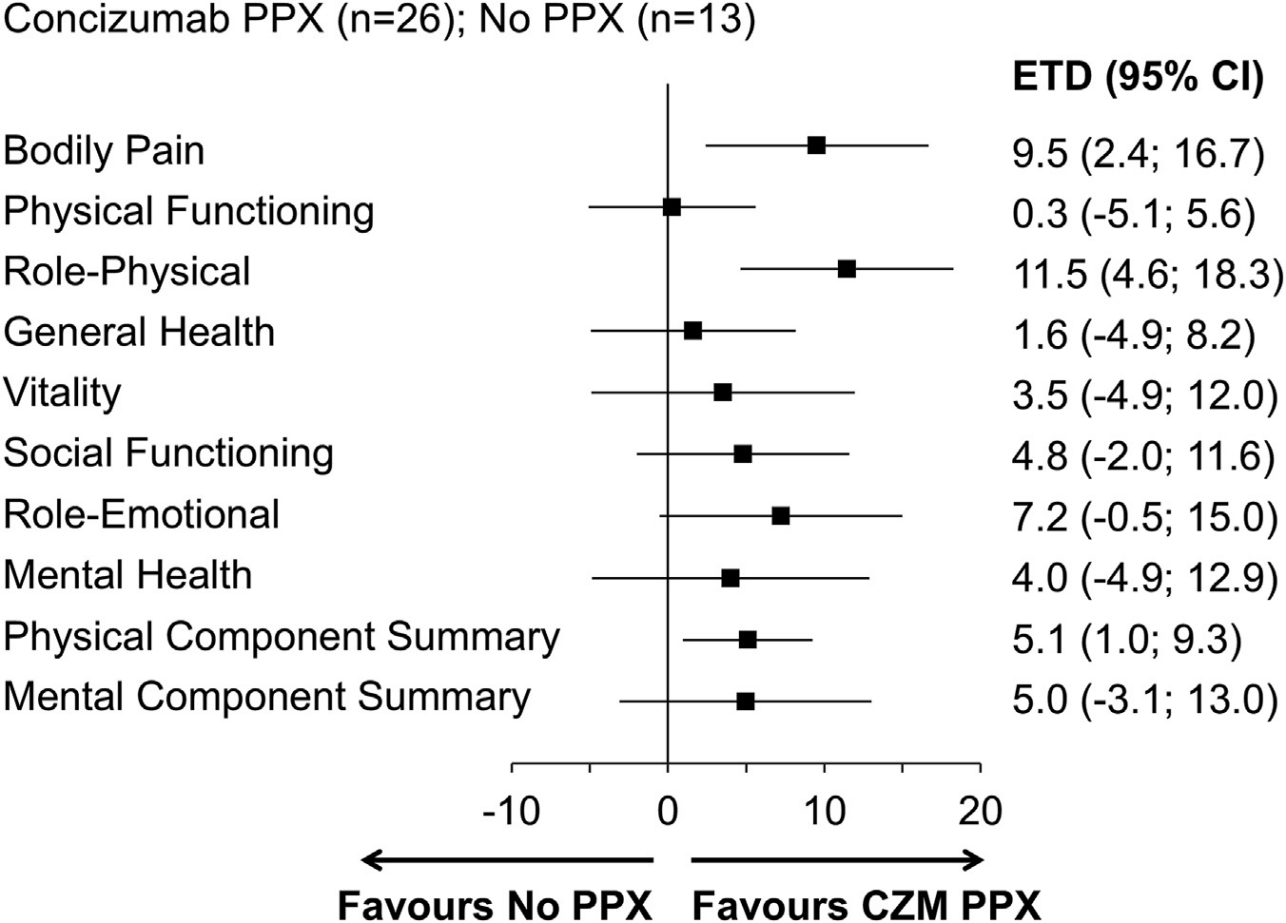
Health-related quality of life (36-item short-form health survey version 2.0 [SF-36v2]) in patients with hemophilia A or B without inhibitors receiving no prophylaxis (PPX; arm 1) or concizumab (CZM) PPX (arm 2). Estimated treatment difference (ETD) in SF-36v2 domain scores and component summary scores at week 24 in patients receiving CZM PPX vs no PPX is shown. Change from baseline to week 24 for patients in arms 1 and 2 was analyzed with mixed model for repeated measures, with treatment, type of hemophilia, and bleeding frequency prior to screening as factors and the baseline value as a covariate. Higher scores in the SF-36v2 correspond to better health-related quality of life.

Of the 148 patients randomized or allocated to arms 1 to 4 after treatment restart, 104 and 120 patients responded to the SF-36v2 questionnaire at baseline and at week 24, respectively (Supplementary Table S1). Mean “bodily pain” score increased from baseline to week 24 for patients in arms 2 to 4, whereas it decreased for patients in arm 1. Mean “physical functioning” score increased from baseline to week 24 for patients in all arms. Mean scores in the remaining domains at week 24 were generally higher than baseline in arms 2 to 4 and lower than baseline in arm 1 (Supplementary Figure S1). The estimated treatment difference (ETD) between patients in arm 1 and arm 2 for the key endpoints, change in “bodily pain” and “physical functioning” from baseline to week 24, was 9.5 points (95% CI, 2.4 to 16.7) and 0.3 points (95% CI, −5.1 to 5.6), respectively (Figure 2). The ETD for all SF-36v2 domains favored concizumab (Figure 2). The ETD by hemophilia subtype is presented in Supplementary Figure S2.

**Figure 3:**
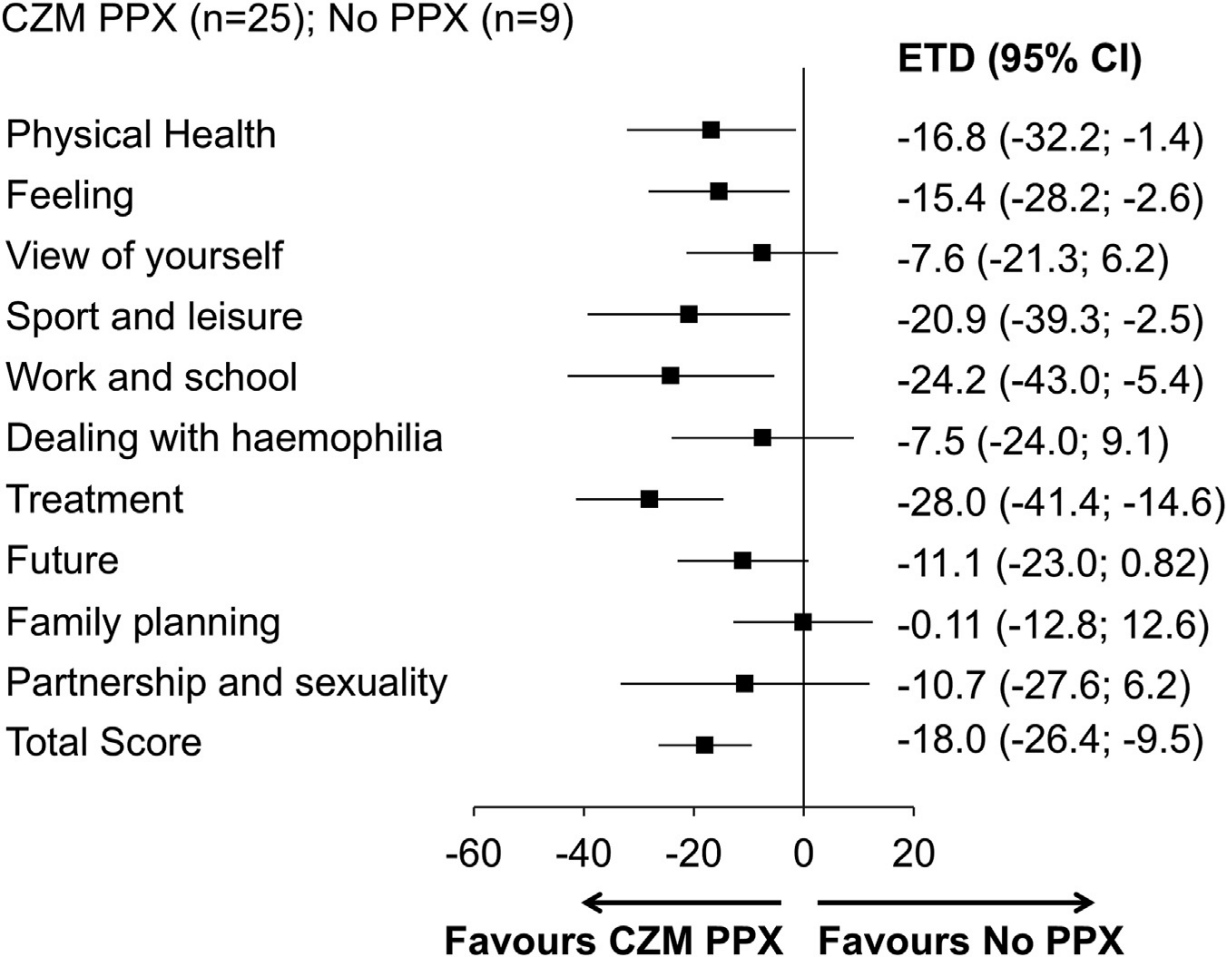
Health-related quality of life (Haemophilia Quality of Life Questionnaire for Adults [Haem-A-QoL]) in patients with hemophilia A or B without inhibitors receiving no prophylaxis (PPX; arm 1) or concizumab (CZM) PPX (arm 2). Estimated treatment difference (ETD) in Haem-A-QoL domain scores and “total score” at week 24 in patients receiving CZM PPX vs no PPX is shown. Change from baseline to week 24 for patients in arms 1 and 2 was analyzed with mixed model for repeated measures, with treatment, type of hemophilia, and bleeding frequency prior to screening as factors and the baseline value as a covariate. Lower scores in the Haem-A-QoL correspond to better health-related quality of life.

In arms 1 to 4, 99 and 71 patients completed the Haem-A-QoL questionnaire at baseline and at week 24, respectively; fewer patients completed the questions or selected “not applicable” in the domains “sports and leisure,” “work and school,” “family planning,” and “partnership and sexuality” (Supplementary Table S1). Mean “total score” and “physical health” scores decreased in arms 2 to 4, while they increased in arm 1 from baseline to week 24. Mean scores in the remaining Haem-A-QoL domains at week 24 were generally lower than at baseline in arms 2 to 4 and higher than at baseline in arm 1 (Supplementary Figure S3). The ETD at week 24 between patients in arm 1 and arm 2 was −18.0 points (95% CI, −26.4 to −9.5) for “total score” and −16.8 points (95% CI, −32.2 to −1.4) for “physical health” (Figure 3). The ETD for all Haem-A-QoL domains favored concizumab over no prophylaxis (Figure 3). The ETD by hemophilia subtype is presented in Supplementary Figure S4.

#### 3.2.2 PROMIS

Of the 148 patients randomized or allocated to arms 1 to 4 after treatment restart, 98 and 117 patients responded to the PROMIS numeric rating scale v1.0 pain intensity 1a questionnaire at baseline and at week 24, respectively (Supplementary Table S1). Mean PROMIS pain intensity score estimates at week 24 were 3.4 (95% CI, 1.5 to 5.2) and 2.8 (95% CI, 1.7 to 3.8) for arms 1 and 2, respectively (Supplementary Table S2). Mean change from baseline score estimates at week 24 were −0.4 (95% CI, −2.3 to 1.5) and −1.0 (95% CI, −2.0 to 0.1) for arms 1 and 2, respectively. The ETD at week 24 between arms 1 and 2 was −0.6 (95% CI, −2.7 to 1.6). A total of 67 and 79 of the 148 patients in arms 1 to 4 responded to the PROMIS short-form v2.0 upper extremity 7a questionnaire at baseline and at week 24, respectively (Supplementary Table S1). Mean PROMIS upper extremity functioning score estimates at week 24 were 43.4 (95% CI, 37.5 to 49.4) and 46.5 (95% CI, 42.8 to 50.1) for arms 1 and 2, respectively (Supplementary Table S3). Mean change from baseline score estimates at week 24 were 0.7 (95% CI, −5.2 to 6.7) and 3.8 (95% CI, 0.1 to 7.4) for arms 1 and 2, respectively. The ETD at week 24 between arms 1 and 2 was 3.0 (95% CI, −4.0 to 10.0).

#### 3.2.3 PGI-S and PGI-C

At week 24, 102 patients in arms 2 to 4 and 13 patients in arm 1 completed the PGI-S and PGI-C questionnaires. Of those who completed the PGI-S questionnaire, 28 (27.5%) patients in arms 2 to 4 reported their level of physical functioning as “very good,” 47 (46.1%) as “good,” 21 (20.6%) as “fair,” 4 (3.9%) as “poor,” and 2 (2.0%) as “very poor.” In arm 1, 5 (38.5%) patients reported their level of physical functioning as “good,” 4 (30.8%) as “fair,” 2 (15.4%) as “poor,” 1 (7.7%) as “very good,” and 1 (7.7%) as “very poor.” For the PGI-C questionnaire, 36 (35.3%) patients in arms 2 to 4 who completed the questionnaire reported the overall change in their level of physical functioning as “very much better,” 24 (23.5%) as “moderately better,” 22 (21.6%) as “a little better,” 17 (16.7%) as “no change,” 2 (2.0%) as “a little worse,” and 1 (1.0%) as “very much worse.” In arm 1, 11 (84.6%) of patients reported “no change” in their level of physical functioning since starting treatment, whereas 1 (7.7%) patient reported the change as “very much better” and 1 (7.7%) patient as “a little better.”

#### 3.2.4 Treatment burden

**Figure 4:**
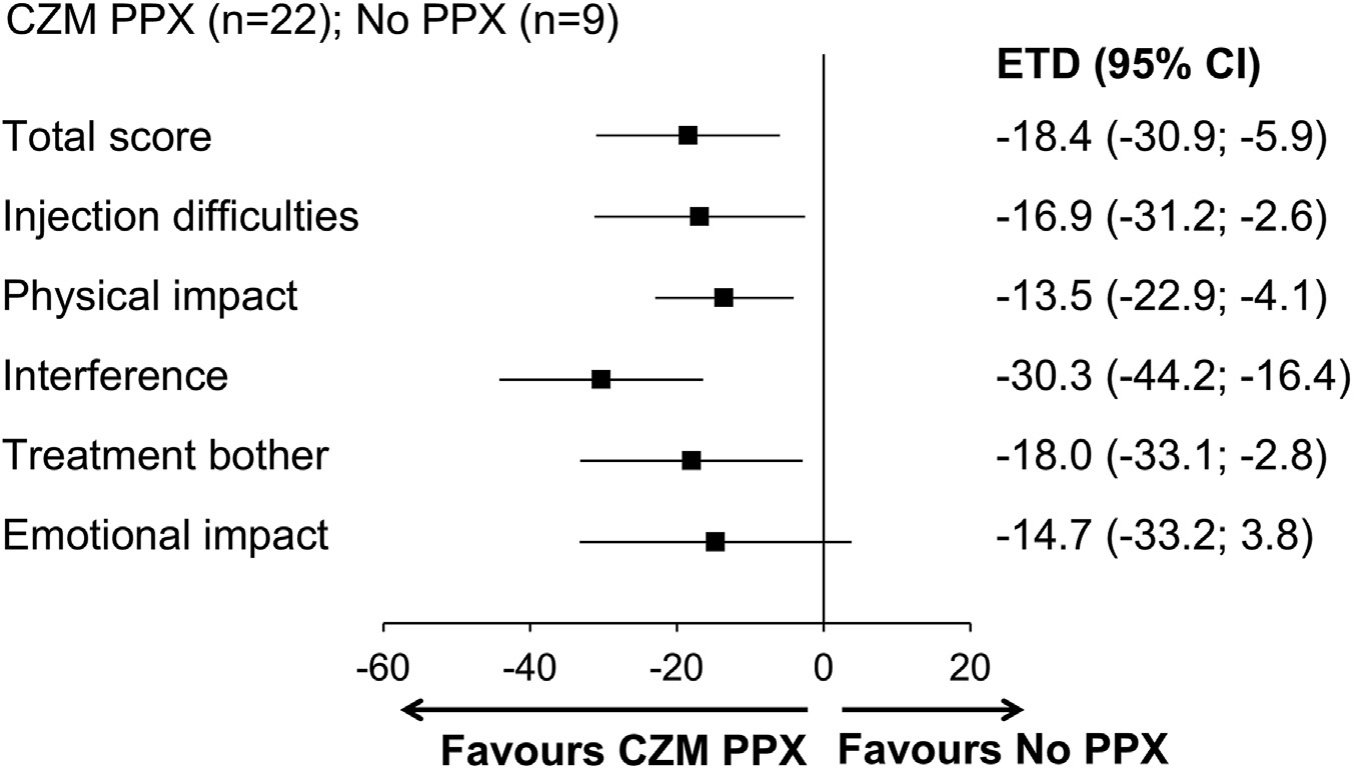
Treatment burden (Hemophilia Treatment Experience Measure [Hemo-TEM]) in patients with hemophilia A or B without inhibitors receiving no prophylaxis (PPX; arm 1) or concizumab (CZM) PPX (arm 2). Estimated treatment difference (ETD) in Hemo-TEM “total score” and domain scores at week 24 in patients receiving CZM PPX vs no PPX are shown. Change from baseline to week 24 for patients in arms 1 and 2 was analyzed with analysis of covariance, with treatment, type of hemophilia, and bleeding frequency prior to screening as factors and the baseline value as a covariate. Lower scores in the Hemo-TEM correspond to lower treatment burden.

Of the 148 patients randomized or allocated to arms 1 to 4 after treatment restart, 99 and 117 patients responded to the Hemo-TEM questionnaire at baseline and at week 24, respectively (Supplementary Table S1). Mean Hemo-TEM “total score” decreased in all arms from baseline to week 24. Mean scores in all other domains generally decreased in all arms from baseline to week 24, except for “interference” and “treatment bother” in arm 1, for which increased scores were observed (Supplementary Figure S5). The ETD at week 24 between patients in arm 1 and arm 2 was −18.4 points (95% CI, −30.9 to −5.9) for “total score” (Figure 4). The ETD for all Hemo-TEM domains favored concizumab over no prophylaxis (Figure 4). The ETD by hemophilia subtype is presented in Supplementary Figure S6.

#### 3.2.5 Treatment preference

**Figure 5:**
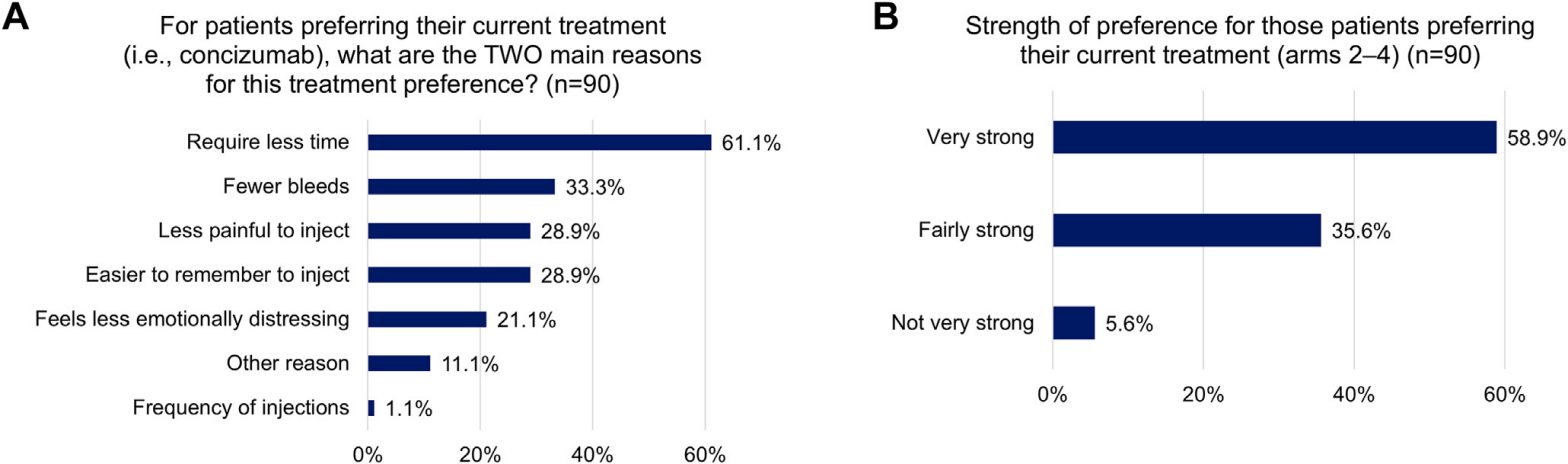
Treatment preferences (Haemophilia Patient Preference Questionnaire) in patients with hemophilia A or B without inhibitors receiving concizumab prophylaxis (arms 2 to 4). Treatment preference results from the Haemophilia Patient Preference Questionnaire in patients with hemophilia A or B without inhibitors receiving concizumab prophylaxis in arms 2 to 4 who responded to the questionnaire (*n* = 102 from a total of 127 patients). Patients who preferred their current treatment (ie, concizumab, *n* = 90) were asked to provide (A) the 2 main reasons for their preference, and (B) indicate the strength of their preference.

The H-PPQ was used to analyze treatment preference for patients. Of 127 patients in arms 2 to 4 who received concizumab, 25 (19.7%) patients did not respond to the questionnaire, 90 (70.9%) patients preferred concizumab, 10 (7.9%) had no preference, and 2 (1.6%) preferred their previous treatment. When asked to select the 2 main reasons for their treatment preference, the most common reasons patients selected for preferring concizumab were “require less time” (61.1%), “fewer bleeds” (33.3%), “less painful to inject” (28.9%), and “easier to remember to inject” (28.9%) (Figure 5A).Treatment preference for concizumab was “very strong” in 53/90 (58.9%) patients, “fairly strong” in 32/90 (35.6%) patients and “not very strong” in 5/90 (5.6%) patients (Figure 5B). H-PPQ results from a subset of arm 4 patients who had been on stable prophylaxis previously in the explorer6 observational study were consistent with the full study population, with most patients preferring concizumab over their previous treatment, and the most common reasons for concizumab preference being “require less time” and “easier to remember to inject” (Supplementary Figure S7).

### 3.3 MWPC

A post hoc, exploratory analysis showed that 45.5% of patients in arm 2 achieved an increase in SF-36v2 score greater than or equal to the MWPC threshold of 3.16 in the “physical functioning” domain, compared with 44.4% of patients in arm 1. A total of 41.2% of patients receiving concizumab (arm 2) achieved a decrease in Haem-A-QoL score greater than or equal to the MWPC threshold of 17.66 for “physical health,” compared with the 25.0% of patients on no prophylaxis (arm 1).

## 4 Discussion

Results from the phase 3 explorer8 study demonstrated the efficacy and safety of daily subcutaneous concizumab prophylaxis vs on-demand treatment in patients with HA/HB without inhibitors [24]. PRO results can provide further insights into the potential benefits of concizumab through patients’ perspectives on HRQoL and treatment experience. PRO results in patients with HA/HB without inhibitors (explorer8) were consistent with those in patients with HA/HB with inhibitors (explorer7), where concizumab was associated with improved HRQoL in some domains, lower treatment burden, and patient preference over no prophylaxis [33].

### 4.1 Effect of concizumab on HRQoL

Trends favoring concizumab over no prophylaxis were observed in the SF-36v2 and Haem-A-QoL results in patients with HA/HB, as observed in the ETD of change in “bodily pain” and change in “physical functioning” from baseline to week 24 in the SF-36v2 questionnaire, along with the Haem-A-QoL “total score” and the “physical health” domain score. Results from PGI-S and PGI-C showed that a higher proportion of patients receiving concizumab prophylaxis reported improvements in physical functioning compared with patients receiving no prophylaxis. PROMIS pain intensity and upper extremity score estimates also favored concizumab over no prophylaxis. When evaluating MWPC, SF-36v2 physical functioning domain results were similar between patients randomized to no prophylaxis and those to concizumab prophylaxis, while the proportion of patients who achieved the MWPC threshold in Haem-A-QoL physical health domain was higher for patients randomized to concizumab prophylaxis than for those randomized to no prophylaxis.

### 4.2 Effect of concizumab on treatment burden

Based on the Hemo-TEM results, patients with HA/HB in explorer8 receiving once-daily subcutaneous concizumab prophylaxis experienced a reduction in treatment burden from baseline to week 24. These results are consistent with those observed in patients with HA/HB with inhibitors (explorer4, phase 2, NCT03196284; and explorer7, phase 3) and patients with severe HA without inhibitors (explorer5, phase 2, NCT03196297), which demonstrated a reduction in treatment burden for patients receiving concizumab prophylaxis [33,35]. The subcutaneous mode of delivery of concizumab likely contributes to the reduced treatment burden as it is less invasive than intravenous administration.

### 4.3 Patients’ treatment preference

Results from the H-PPQ demonstrated that most patients in this study expressed a preference for concizumab prophylaxis compared with their previous treatment, which aligned with the explorer7 results in people with hemophilia with inhibitors [23]. The ease of administration and reduced pain levels of injections appeared to be the main reasons for preferring concizumab. It is worth noting that concizumab is administered subcutaneously via a pen-injector with a short, thin, 4-mm, 32-guage needle, which may be associated with reduced pain levels. In addition, a previous study showed that people with hemophilia and hemophilia caregivers found the concizumab pen-injector “easy” or “very easy to use,” noting that its use could be learned quickly (<10 minutes on average) and involved short preparation and injection time (<2 minutes on average) [36].

### 4.4 Limitations

Potential bias may be present due to some aspects of the study design. The explorer8 study was open-label, which may have introduced bias in the PRO results as patients were aware of their treatment allocation. Moreover, patients in arms 3 and 4 were not randomized, which may be a potential source of selection bias.

Missing data limited the interpretation of PRO results in this study. The CIs were also relatively large in the PRO results. The main reasons for missing data included lack of confirmation of visits in the study management system or patients not completing the required practice diary at their initial visit, which led to a loss of baseline values. As such, the MMRM statistical approach was selected for data analysis, only including patients who had completed a questionnaire at both the baseline visit and at ≥1 visits after baseline. Patient participation in PRO questionnaires remains an aspect requiring further attention to mitigate the effects of missing data in future studies.

MWPC was not a predefined endpoint but a post hoc and exploratory analysis; therefore, results should be interpreted with caution as the study was not designed for this purpose.

## 5 Conclusion

PRO data collected in this study provided additional support for once-daily subcutaneous concizumab prophylaxis compared with no prophylaxis as a treatment option for patients with HA/HB.
