# Supplementary material for: Concizumab prophylaxis in people with hemophilia A or B without inhibitors: patient-reported outcome results from the phase 3 explorer8 study
Source: Res Pract Thromb Haemost. 2025 Feb 20;9(2):102705. doi: 10.1016/j.rpth.2025.102705 (PMC11957488; doi:10.1016/j.rpth.2025.102705)
Supplement: Supplementary Material [file mmc1.docx]

# Title:

Concizumab prophylaxis in patients with haemophilia A or B without inhibitors: Patient‑reported outcome results from the phase 3 explorer8 study

**Authors**:

Pantep Angchaisuksiri,^1*^ Sylvia von Mackensen,^2^ Shashikant Apte,^3^ Gary Benson,^4^ Hermann Eichler,^5^ Amy Findley,^6^ Tadashi Matsushita,^7^ Camila M Mazini Tavares,^6^ Morten Puggaard Ravn,^6^ Jameela Sathar,^8^ Laura Villarreal Martinez,^9^ Guy Young^10^

**Affiliations**:

*^1^Division of Haematology, Department of Medicine, Ramathibodi Hospital, Mahidol University, Bangkok, Thailand;*

*^2^Department of Medical Psychology, University Medical Centre Hamburg-Eppendorf, Hamburg, Germany;*

*^3^Department of Haematology, Sahyadri Specialty Hospitals, Pune, India;*

*^4^Department of Haematology, Belfast Health and Social Care Trust, Belfast, Northern Ireland, UK;*

*^5^Institute of Clinical Haemostaseology and Transfusion Medicine, Saarland University and University Hospital, Homburg, Germany;*

*^6^Novo Nordisk A/S, Søborg, Denmark;*

*^7^Department of Transfusion Medicine, Nagoya University Hospital, Nagoya, Japan;*

*^8^Department of Haematology, Ampang Hospital, Kuala Lumpur, Malaysia;*

*^9^Department of Haematology, Dr. José Eleuterio González Monterrey University Hospital, Monterrey, Nuevo León, México*

*^10^Hemostasis and Thrombosis Centre, Children’s Hospital of Los Angeles, Keck School of Medicine, University of Southern California, Los Angeles, CA, USA.*

**Trial registration:** NCT04082429

# Supplementary methods

## Exclusion criteria for the explorer8 study:

- Known or suspected hypersensitivity to any constituent of the trial product or related products.
- Previous participation in this trial (defined as signed informed consent, although this did not apply for patients who were screen-failed at sponsor discretion due to the trial pause).
- Participation in any clinical trial of an approved or non-approved investigational medicinal product within 5 half-lives or 30 days from screening, whichever is longer (not applicable for patients from explorer5; NCT03196297).
- Platelets ≤100 x 10^9^/L at screening.
- Fibrinogen below laboratory lower normal limit at screening.
- Hepatic dysfunction defined as alanine aspartate aminotransferase and/or alanine aminotransferase >3 times the upper limit of normal combined with total bilirubin >1.5 times the upper limit of normal at screening.
- Renal impairment defined as estimated Glomerular Filtration Rate ≤30 mL/min/1.73 m^2^ for serum creatinine measured at screening.
- Known inherited or acquired coagulation disorder other than congenital haemophilia.
- History of thromboembolic disease (including arterial and venous thrombosis, including myocardial infarction, pulmonary embolism, cerebral infarction/thrombosis, deep vein thrombosis, other clinically significant thromboembolic events and peripheral artery occlusion).
- Current clinical signs of or treatment for thromboembolic disease (including arterial and venous thrombosis such as myocardial infarction, pulmonary embolism, cerebral infarction/thrombosis, deep vein thrombosis, other clinically significant thromboembolic events and peripheral artery occlusion).
- Patients who in the judgement of the investigator were considered at high risk of thromboembolic events (thromboembolic risk factors could include, but are not limited to, hypercholesterolemia, diabetes mellitus, hypertension, obesity, smoking, family history of thromboembolic events, arteriosclerosis, other conditions associated with increased risk of thromboembolic events).
- A known systemic inflammatory condition requiring systemic treatment at screening.
- Treatment with emicizumab within 180 days before screening.
- Presence of confirmed inhibitor ≥0.6 Bethesda Unit (BU) at screening.
- Known history of inhibitors ≥0.6 BU in the last 5 years according to the medical records.
- Any disorder, except for conditions associated with haemophilia, which in the investigator’s opinion might jeopardize patient’s safety or compliance with the protocol.

## Trial pause

- Concizumab treatment was paused due to five thromboembolic events in three patients in the phase 3 explorer7 (NCT04083781) and explorer8 studies.
  - Two patients from explorer8 experienced four thromboembolic events (deep vein thrombosis, pulmonary embolism, superficial vein thrombosis in one patient and acute myocardial infarction in the other patient).
  - One patient from explorer7 experienced non-fatal thromboembolic events (renal infarction).
  - Consequently, the Food and Drug Administration issued a clinical hold.^1,2^
- All three patients who experienced the non-fatal thromboembolic events were found to have different thromboembolic risk factors at baseline.
  - The three patients had also used concomitant haemostatic medication on the day of (and in two cases during the days preceding) onset of the event (rFVIIa in the patient with haemophilia B with inhibitors from explorer7 and FVIII in the patients with haemophilia A from explorer8), at a high dose or with frequent dosing. The concizumab exposure levels in 2 out of 3 patients were at the higher end of the exposure range observed in the phase 2 and phase 3 trials.
  - All five thromboembolic events were judged as possibly or probably related to concizumab prophylaxis by the investigator and reported as ‘recovered or recovering’, except one event (renal infarct) which was reported as ‘recovered/resolved with sequalae’, as the patient will have ongoing scarring, secondary to the thromboembolism, despite normal renal function.
- After thorough investigations of all available data, concizumab treatment in the trials were resumed, with a risk mitigation strategy in place. Primary mitigation actions included:
  - Patients must always contact the trial site before treating a bleeding episode (i.e., when they have a suspected bleed).
  - A new guidance for the treatment of mild and moderate bleeding episodes, with specific guidance for use of the lowest dose of factor product or bypassing agent while on concizumab prophylaxis.
- Additional mitigations actions included:
  - A new concizumab dosing regimen (an initial loading dose of 1.0 mg/kg on day 1, followed by a daily dose of 0.20 mg/kg from day 2).
    - The previous dosing regimen was an initial loading dose of 1 mg/kg, followed by a daily dose of 0.25 mg/kg from day 2.
    - A maintenance dose setting step was included, as described in the main manuscript text.
    - Exposure to concizumab was measured using an anti-concizumab enzyme-linked immunosorbent assay (ELISA).
  - Elective major surgery was no longer allowed.
  - The trial stopping rule was modified to state that urgent evaluation was needed by the Novo Nordisk safety committee and the external independent Data Monitoring Committee in the case of one significant thrombotic event (instead of 2), disseminated intravascular coagulation, thrombotic microangiopathy or death of a trial patient that was potentially related to the trial product.
- Exclusion criteria remained unchanged, as no common risk factors were identified among the three patients who experienced thromboembolic events.

# **Supplementary Table S1.** Patient-reported outcome questionnaires and number of respondents

|  | **Randomised (n)** | | | | **Non-randomised (n)** | | | |
| --- | --- | --- | --- | --- | --- | --- | --- | --- |
|  | **Arm 1 (no prophylaxis)** | | **Arm 2 (concizumab prophylaxis)** | | **Arm 3^e^ (concizumab prophylaxis)** | | **Arm 4^f^ (concizumab prophylaxis)** | |
| Full analysis set | 21 | | 42 | | 9 | | 76 | |
| Full analysis set and Analysis data set | 21 | | 42 | | 9 | | 76 | |
| Completed 56 weeks of concizumab treatment on new regimen | ^-^ | | 39 | | 7 | | 69 | |
| Completed at CACO | 21 | | 39 | | 7 | | 70 | |
|  | **Baseline** | **Week 24** | **Baseline** | **Week 24** | **Baseline** | **Week 24** | **Baseline** | **Week 24** |
| **SF-36v2** | 15 | 13 | 29 | 32 | 8 | 7 | 52 | 68 |
| **Haem-A-QoL^a^** | 10 | 8 | 25 | 17 | 8 | 5 | 48 | 41 |
| **Hemo-TEM** | 14 | 13 | 29 | 32 | 7 | 7 | 49 | 65 |
| **H-PPQ^b^** |  |  |  | 32 |  | 7 |  | 63 |
| **PROMIS-Pain** | 13 | 13 | 29 | 32 | 8 | 7 | 48 | 65 |
| **PROMIS-UE** | 10 | 10 | 21 | 23 | 5 | 4 | 31 | 42 |

148 patients with HA/HB were randomly assigned or allocated to arms 1–4 after treatment restart in explorer8. The n used for statistical analyses varied by questionnaire.

^a^Only for patients ≥17 years of age; fewer patients completed the questions or selected ‘not applicable’ in the domains ‘sport and leisure’ (arm 1: n=8 at baseline and Week 24; arm 2: n=23 at baseline, n=13 at Week 24; arm 3: n=6 at baseline, n=1 at Week 24; arm 4: n=39 at baseline, n=32 at Week 24), ‘work and school’ (arm 1: n=7 at baseline, n=5 at Week 24; arm 2: n=23 at baseline, n=17 at Week 24; arm 3: n=6 at baseline, n=3 at Week 24; arm 4: n=45 at baseline, n=39 at Week 24), ‘family planning’ (arm 1: n=5 at baseline and Week 24; arm 2: n=19 at baseline, n=12 at Week 24; arm 3: n=4 at baseline, n=2 at Week 24; arm 4: n=34 at baseline, n=25 at Week 24); fewer patients completed the questions in the domain ‘partnership and sexuality’ (arm 1: n=10 at baseline, n=7 at Week 24; arm 2: n=25 at baseline, n=16 at Week 24; arm 3: n=8 at baseline, n=5 at Week 24; arm 4: n=48 at baseline, n=41 at Week 24). ^b^H-PPQ was only administered at Week 24 to patients receiving concizumab in arms 2–4.

CACO, confirmatory analysis cut-off; Haem-A-QoL, Haemophilia Quality of Life Questionnaire for Adults; Hemo-TEM, Hemophilia Treatment Experience Measure; H-PPQ, Haemophilia Patient Preference Questionnaire; PROMIS, Patient-Reported Outcomes Measurement Information System; SF-36v2, 36-item Short-Form Health Survey version 2; UE, upper extremities

# Supplementary Table S2. PROMIS Numeric Rating scale v1.0 - Pain Intensity 1a questionnaire responses from patients receiving no prophylaxis (arm 1) or concizumab prophylaxis (arm 2)

|  | **Arm** **1 (no prophylaxis)** | **Arm** **2 (concizumab prophylaxis)** |
| --- | --- | --- |
| **N contributing to analysis** | 11 | 26 |
| Mean score estimates at Week 24 (95% CI) | 3.4 (1.5; 5.2) | 2.8 (1.7; 3.8) |
| Mean change from baseline score estimates at Week 24 (95% CI) | -0.4 (-2.3; 1.5) | -1.0 (-2.0; 0.1) |
| Difference estimate at Week 24 (95% CI) | -0.6 (-2.7; 1.6) | |

Average pain intensity experienced in the past seven days from 0 (no pain) to 10 (worst imaginable pain) was estimated using a mixed model for repeated measures (MMRM) for patients with results at baseline and ≥1 visits post-baseline. Higher scores indicate greater pain intensity.

CI, confidence interval; PROMIS, patient-reported outcome measurement information system

# Supplementary Table S3. PROMIS Short Form v2.0 Upper Extremity 7a questionnaire responses from patients receiving no prophylaxis (arm 1) or concizumab prophylaxis (arm 2)

|  | **Arm 1  (no prophylaxis)** | **Arm 2  (concizumab prophylaxis)** |
| --- | --- | --- |
| **N contributing to analysis** | 8 | 17 |
| Mean score estimates at Week 24 (95% CI) | 43.4 (37.5; 49.4) | 46.5 (42.8; 50.1) |
| Mean change from baseline score estimates at Week 24 (95% CI) | 0.7 (-5.2; 6.7) | 3.8 (0.1; 7.4) |
| Difference estimate at Week 24  (95% CI) | 3.0 (-4.0; 10.0) | |

Higher scores in the PROMIS Short Form v2.0 Upper Extremity 7a indicate a higher level of physical functioning, which was estimated using a mixed model for repeated measures (MMRM) for patients with results at baseline and ≥1 visits post-baseline. Higher scores indicate a higher level of physical functioning.

CI, confidence interval; PROMIS, patient-reported outcome measurement information system

# Supplementary Figure S1. Mean SF-36v2 scores at baseline and at Week 24 for patients with HA/HB

**
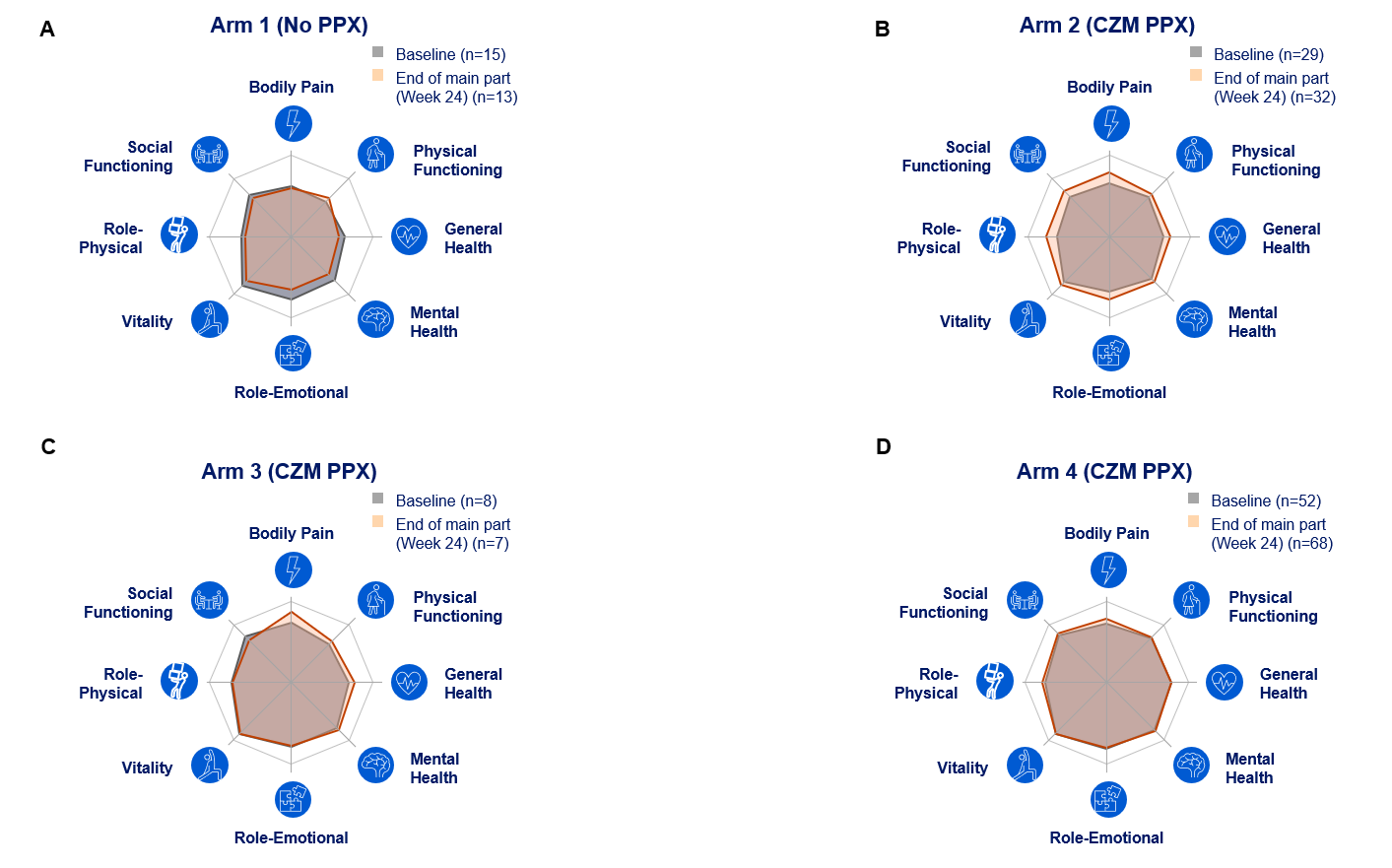
**

The radar plots show mean SF-36v2 scores for each domain at baseline and at Week 24. Higher SF-36v2 scores indicate better HRQoL.
CZM, concizumab; HRQoL, health-related quality of life; PPX, prophylaxis; SF-36v2, 36-item Short-Form Health Survey version 2

# **Supplementary Figure S2**. Health-related quality of life (SF‑36v2) in patients with (A) HA and (B) HB receiving no prophylaxis (arm 1) or concizumab prophylaxis (arm 2)


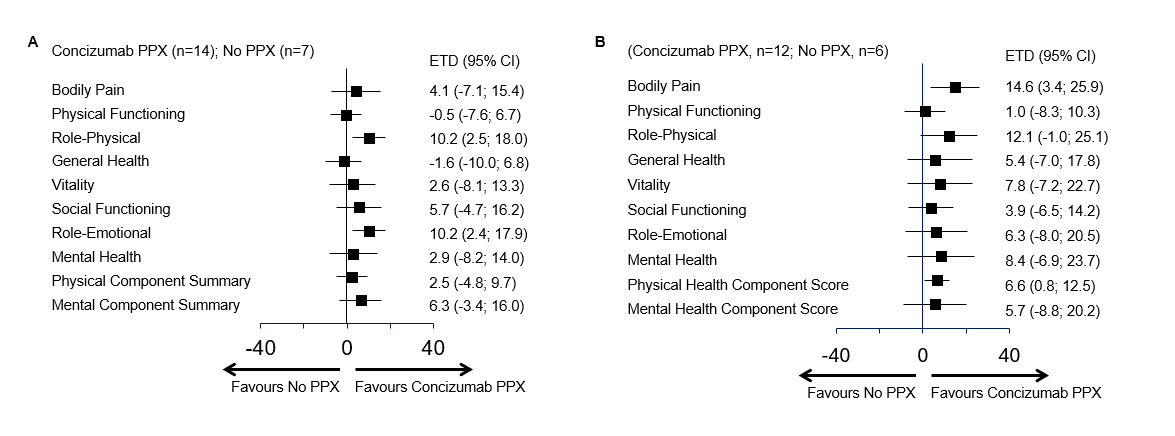


The ETD was determined after 24 weeks for all SF-36v2 categories between patients with HA or HB on once-daily CZM PPX or no PPX using a MMRM. In the SF-36v2 questionnaire, higher scores indicate better HRQoL.
CI, confidence interval; CZM, concizumab; ETD, estimated treatment difference; HA, haemophilia A; HB, haemophilia B; HRQoL, health-related quality of life; MMRM, mixed model for repeated measures; PPX, prophylaxis; SF-36v2, 36‑item Short‑Form Health Survey version 2

#
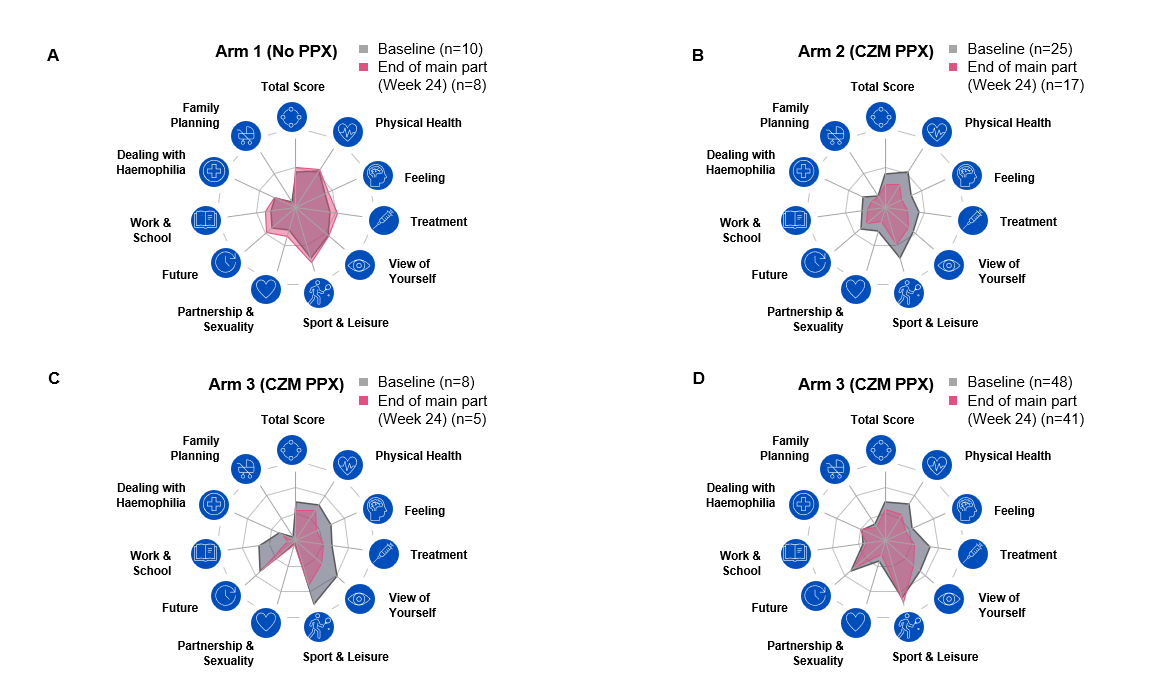
Supplementary Figure S3. Mean Haem‑A‑QoL scores at baseline and at Week 24 for patients with HA/HB

The radar plots show mean Haem-A-QoL scores for each domain at baseline and at Week 24, lower Haem-A-QoL scores correspond to better HRQoL.
CZM, concizumab; Haem‑A‑QoL, Haemophilia Quality of Life Questionnaire for Adults; HRQoL, health-related quality of life; PPX, prophylaxis

# **Supplementary Figure S4.** Health-related quality of life (Haem‑A‑QoL) in patients with (A) HA and (B) HB receiving no prophylaxis (arm 1) or concizumab prophylaxis (arm 2)


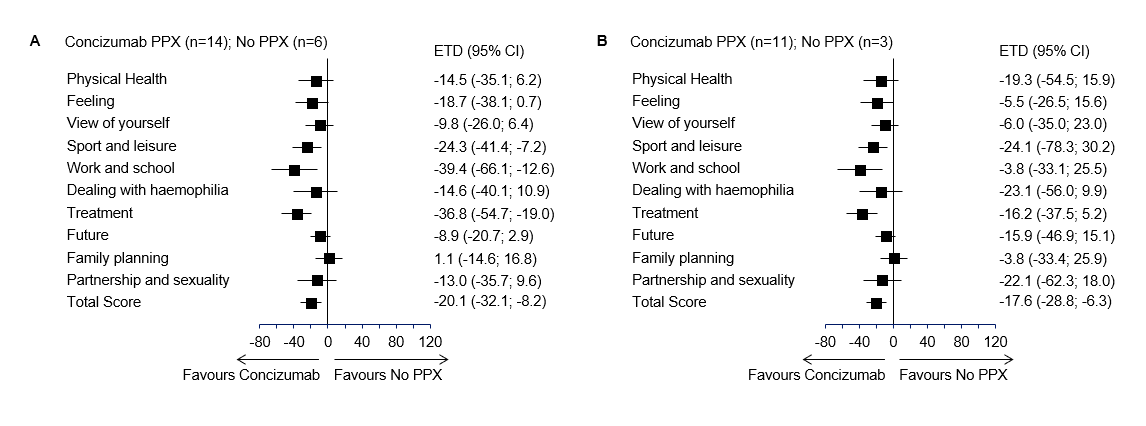


The ETD was determined after 24 weeks for all Haem-A-QoL domains between patients with HA or HB on once-daily CZM PPX or no PPX, using a MMRM. In the Haem A QoL questionnaire, lower scores correspond to better HRQoL. Fewer patients were analysed in the domains “sport and leisure” (HA n=5 no PPX / n=13 CZM PPX; HB n=2 no PPX / n=9 CZM PPX); “work and school” (HA n=4 no PPX / n=12 CZM PPX; HB n=2 no PPX / n=10 concizumab PPX).

CI, confidence interval; CZM, concizumab; ETD, estimated treatment difference; Haem-A-QoL, Haemophilia Quality of Life Questionnaire for Adults; HA, haemophilia A; HB, haemophilia B; HRQoL, health-related quality of life; MMRM, mixed model for repeated measures; PPX, prophylaxis

# Supplementary Figure S5. Mean Hemo-TEM scores at baseline and Week 24 for patients with HA/HB


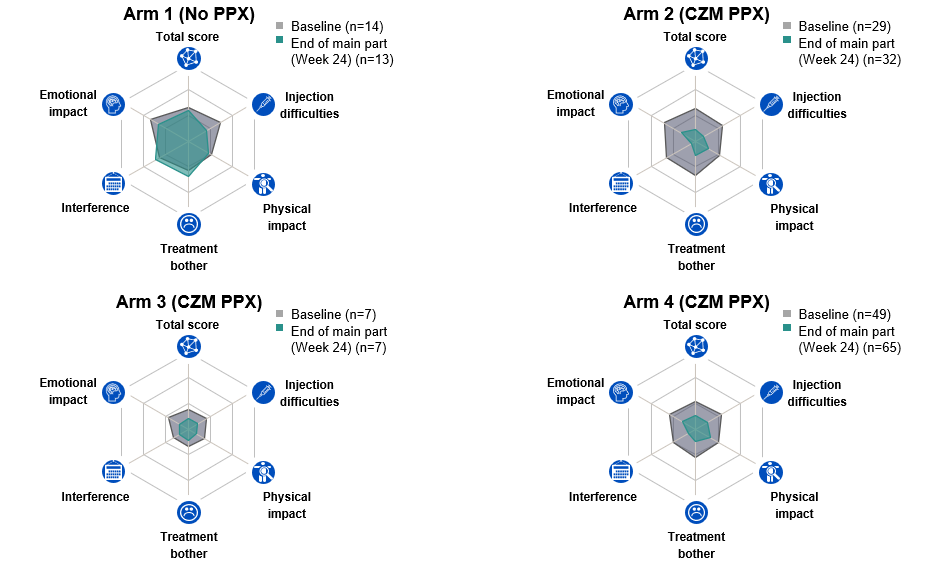


The radar plots show mean Hemo-TEM scores for each domain at baseline and at Week 24, lower Hemo-TEM scores indicate lower treatment burden.
CZM, concizumab; Hemo-TEM, Haemophilia Treatment Experience Measure; PPX, prophylaxis

# Supplementary Figure S6. Treatment burden (Hemo‑TEM) in patients with (A) HA and (B) HB receiving no prophylaxis (arm 1) or concizumab prophylaxis (arm 2)


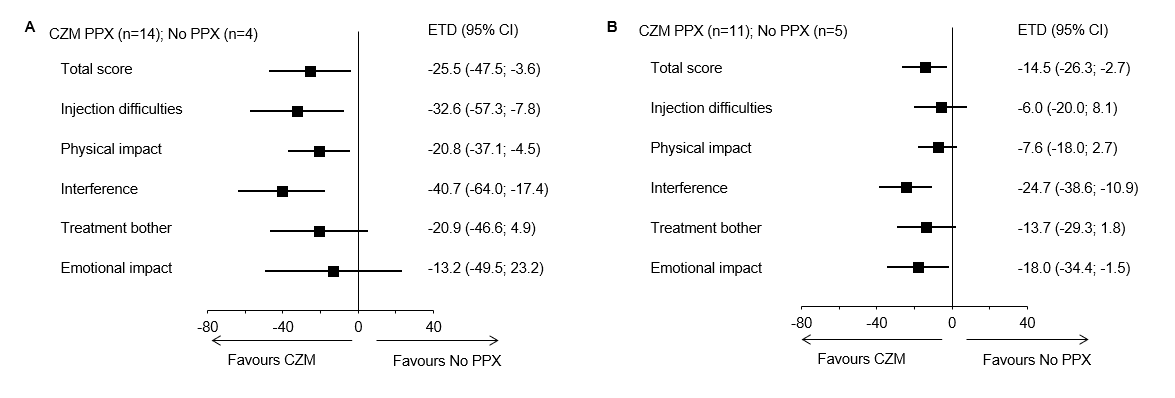


The ETD was determined after 24 weeks for Hemo-TEM domains between patients with HA or HB on once-daily concizumab PPX or no PPX using an ANCOVA model with treatment and bleeding frequency prior to screening as factors, and baseline value as a covariate. Lower scores in all Hemo-TEM domains indicate lower treatment burden.

ANCOVA, analysis of covariance; CI, confidence interval; CZM, concizumab; ETD, estimated treatment difference; HA, haemophilia A; HB, haemophilia B; Hemo-TEM, Haemophilia Treatment Experience Measure; PPX, prophylaxis

# Supplementary Figure S7. Patient-reported treatment preferences in a subset of arm 4 patients with (A) HA and (B) HB who had been on stable prophylaxis previously^a^ (H‑PPQ)


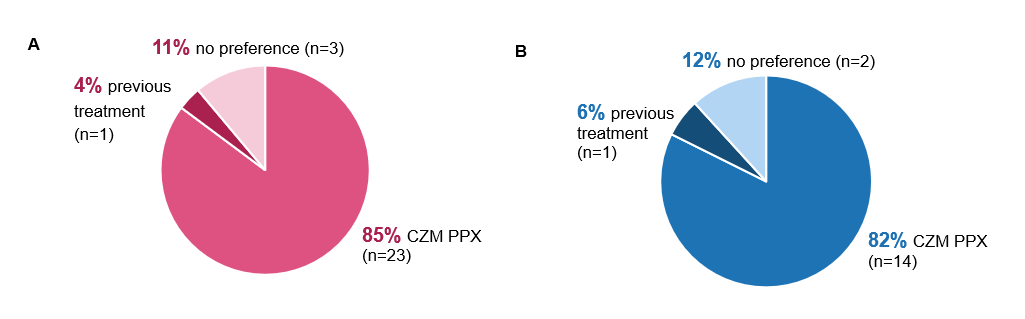


^a^Patients in arm 4 who had been on a stable prophylaxis regimen for at least 24 weeks in explorer6 (NCT03741881) and who entered the maintenance period in explorer8 (NCT04082429).
CZM, concizumab; HA, haemophilia A; HB, haemophilia B; H‑PPQ, Haemophilia Patient Preference Questionnaire; PPX, prophylaxis

# References

1. Matsushita T, Shapiro A, Abraham A, et al. Phase 3 Trial of Concizumab in Hemophilia with Inhibitors. *N Engl J Med*. 2023;389(9):783-794.

2. Seremetis S, Cepo K, Rasmussen J, et al. Risk mitigation strategy for concizumab clinical trials re-initiation after pause due to non-fatal thrombotic events. *Haemophilia*. 2021;27(S2):18-181, Abstract 8.
